# Severely injured patients do not disappear in a pandemic: Incidence and characteristics of severe injuries during COVID-19 lockdown in Finland

**DOI:** 10.1080/17453674.2021.1881241

**Published:** 2021-02-04

**Authors:** Antti Riuttanen, Ville Ponkilainen, Ilari Kuitunen, Aleksi Reito, Joonas Sirola, Ville M Mattila

**Affiliations:** a Department of Orthopedics, Tampere University, Faculty of Medicine and Health Technology and Tampere University Hospital, Tampere;; b Department of Surgery, Central Finland Hospital, Jyväskylä;; c Mikkeli Central Hospital, Mikkeli, University of Eastern Finland, School of Medicine, Kuopio;; d Department of Orthopedics, Tampere University, Faculty of Medicine and Health Technology and Tampere University Hospital, Tampere;; e Kuopio Musculoskeletal Research Unit (KMRU), University of Eastern Finland, Kuopio, University Hospital, Department of Orthopedics, Traumatology and Hand Surgery, Kuopio;; f Department of Orthopedics, Tampere University, Faculty of Medicine and Health Technology and Tampere University Hospital, Tampere, Finland

## Abstract

Background and purpose — COVID-19 lockdowns have resulted in noteworthy changes in trauma admissions. We report and compare the incidence and characteristics of severe injuries (New Injury Severity Score [NISS] > 15) during the COVID-19 lockdown in Finland with earlier years.

Methods — We retrospectively analyzed incidence rate, injury severity scores, injury patterns, and mechanisms of injury of all severely injured patients (NISS >15) in 4 Finnish hospitals (Tampere University Hospital, Kuopio University Hospital, Central Finland Hospital, Mikkeli Central Hospital) during the 11-week lockdown period (March 16–May 31, 2020) with comparison with a matching time period in earlier years (2016–2018). These 4 hospitals have a combined catchment area of 1,150,000 people or roughly one-fifth of the population of Finland.

Results — The incidence rate of severe injuries during the lockdown period was 4.9/10^5^ inhabitants (95% CI 3.7–6.4). The incidence rate of severe injuries during years 2016–2018 was 5.1/10^5^ inhabitants (CI 3.9–6.5). We could not detect a significant incidence difference between the lockdown period and the 3 previous years (incidence rate difference –0.2 (CI –2.0 to 1.7). The proportion of traffic-related accidents was 55% during the lockdown period and 51% during previous years. There were no detectable differences in injury patterns. During the lockdown period, the mean age of patients was higher (53 years vs. 47 years, p = 0.03) and the rate of severely injured elderly patients (aged 70 or more) was higher (30% vs. 16%).

Interpretation — Despite heavy social restrictions, the incidence of severe injuries during the lockdown period was similar to previous years. Notably, a decline in road use and traffic volumes did not reduce the number of severe traffic accidents. Although our data is compatible with a decrease of 2.0 to an increase of 1.7 severely injured patients per 10^5^ inhabitants, we conclude that severely injured patients do not disappear even during pandemic and stabile hospital resources are needed to treat these patients.

On March 16, 2020, the Finnish Government declared a state of emergency in response to the COVID-19 outbreak. All permanent residents were asked to minimize social contacts and to avoid non-essential travel and spending time in public places. Schools and educational institutions were closed down and face-to-face teaching was suspended. In addition, remote working was recommended where possible. Residents aged 70 years and older were told to stay in quarantine-like conditions at home. The capacity of healthcare and social welfare services was increased, and non-urgent activities were reduced (Government Communications Department 2020a). As the highest incidence of COVID-19 was observed in the Uusimaa region, which is the most populous area (population 1,700,000) in Finland, a decision was made to further restrict movement by means of an Emergency Powers Act (Government Communications Department 2020b). The police enforced a lockdown of the Uusimaa region (from March 27 to April 15, 2020), and restricted all non-essential traffic to and from the Uusimaa region. The capital city of Finland, Helsinki, is located in the Uusimaa region. All of the above steps combined resulted in a one-third decline in traffic volumes on the major roads across Finland (Finnish Transport Infrastructure Agency 2020).

**Figure:**
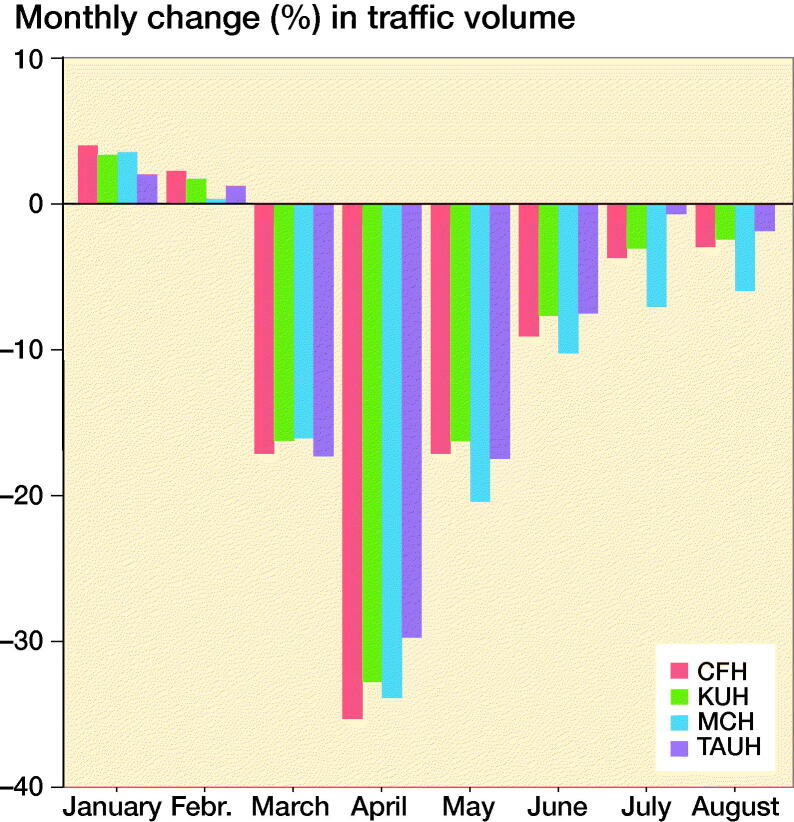
Monthly change in traffic volumes in 2020 compared with 2019 in the 4 hospital regions participating in this study. CFH = Central Finland Hospital region, KUH = Kuopio University Hospital region, MCH = Mikkeli Central Hospital region, TAUH = Tampere University Hospital region.

After the widespread transmission of the disease in Europe, similar regional or national restrictions were set in place in various countries (Lau et al. 2020). The different lockdown procedures implemented have been reported to have had substantial effects on various types of trauma. An early study from New Zealand reported that lockdown resulted in a pronounced 43% reduction in all trauma admissions in a single trauma center (Christey et al. 2020). Similar findings were also reported from the UK, where trauma referrals decreased by nearly 50% (Park et al. 2020). As a result of decreased traffic volumes, the largest reduction was seen in road traffic injuries (Christey et al. 2020). Social distancing and recommendations to avoid large crowds have also increased the amount of time spent at home. Consequently, the place of injury has moved from sportsgrounds to the home (Pellegrini et al. 2020). In France, for example, the rate of domestic hand injuries treated at university trauma hand units doubled, even though the overall rate of upper limb emergencies plummeted (Pinggera et al. 2020).

It is likely that the lockdown procedures imposed in Finland during the COVID-19 pandemic could have changed the incidence and characteristics of injuries in Finland. However, despite multiple reports indicating notable changes in various types of trauma, the information concerning severe injuries is limited. As treatment of both severe injuries and severe COVID-19 infection are resource-consuming, it is important to find out whether the lockdown procedures have had an effect on incidence of severe injuries. Therefore, this study investigates the incidence rate and characteristics of severe injuries (New Injury Severity Score [NISS] > 15) during the lockdown period in Finland and how this rate compares with earlier years.

## Methods

This study is a retrospective cohort study comparing severely injured patient during the lockdown period with severely injured patients treated in the years 2016–2018. The study was conducted in 4 Finnish hospitals (Tampere University Hospital [TAUH], Kuopio University Hospital [KUH], Central Finland Hospital [CFH], and Mikkeli Central Hospital [MCH]) serving a combined population base of approximately 1,150,000 inhabitants (Statistics Finland 2020a). The study population covers roughly one-fifth of the whole population of Finland. To assure adequate coverage we selected 2 major university hospitals, 1 big central hospital, and 1 smaller central hospital. All 4 hospitals operate as the primary trauma care provider in their hospital district providing immediate services in orthopedic surgery, anesthesiology, emergency medicine, radiology, internal medicine, plastic surgery, oral and maxillofacial surgery, pediatrics, and critical care. In addition, TAUH and KUH also serve as tertiary trauma care units providing immediate services in neurosurgery.

The study population consists of severely injured patients treated during the 11-week lockdown period (March 16–May 31, 2020). The data from patients treated in the TAUH region was extracted from TAUH’s Trauma Registry. All trauma patients treated at TAUH meeting the inclusion criteria: (NISS > 15, minimum Maximum Abbreviated Injury Score (MAIS) 3, treated at intensive care unit (ICU) or High Dependency Unit (HDU) have been enrolled prospectively into the TAUH’s Trauma Registry since 2015. Each year, approximately 150 to 200 severely injured patients are enrolled. As TAUH is the only hospital among the participating hospitals with a trauma registry, the data from the other hospitals was retrospectively collected from patient records and coded to match the TAUH Trauma registry. All trauma CTs in these 3 hospitals were retrospectively inspected and both the Injury Severity Score (ISS) and the NISS were calculated. The following inclusion criteria were used: NISS > 15, MAIS ≥ 3, admission through emergency room to further treatment at ICU or HDU.

As the number and characteristics of severe injuries may vary between years, we calculated the reference incidence of severe injuries on the basis of a 3-year average (2016–2018) for all 4 hospitals. The reference incidence for the TAUH region was derived from prospectively collected data from TAUH’s Trauma Register. The reference population for the other 3 hospitals was collected retrospectively with matching inclusion criteria. The 11-week reference period was set out to match the lockdown period in Finland (March 16–May 31, 2020). The incidence rate of severe injuries was calculated based on the population of each hospital area: TAUH (530,000 inhabitants), KUH (250,000), CFH (270,000), and MCH (100,000) (Statistics Finland 2020a).

Mechanisms of injury were extracted from TAUH’s Trauma Registry or from medical records (KUH, CFH, MCH). The causes of injuries were classified with the injury-related 10th revision of the International Classification of Diseases and Related Health Problems (ICD-10) as either traffic- (V01–V99) or non-traffic-related injuries (W00–X59), such as falls and assaults. Traffic-related injuries (V01–V99) included all motor vehicle collisions, such as crashes and driving off the road (V49), injuries caused to pedestrians (V01–V09), motorbike accidents (V28, V29) and injuries that occurred while riding a bicycle (V10–V19). Other traffic-related injuries include miscellaneous injuries, such as collisions with trains (V81) and all-terrain vehicle accidents (V86). Falls (W00–W19) were further divided into high (≥ 3 meters) and low falls (< 3 meters), to better distinguish high-energy falls from low-energy falls. Assaults (X85–Y09) included all forms of physical abuse, including shootings and stabbings. Other accidents included injury mechanisms, such as crush injuries (W23) or injuries caused by falling items (W20), that did not fit in the previous classifications.

Injury patterns were based on the Abbreviated Injury Scale (AIS) (Committee on Medical Aspects of Automotive Safety 1971) with pelvic injuries classified as lower extremity injuries. Minor (AIS1) injuries, such as cuts and bruises, were excluded from the analysis concerning injury patterns because they are seldom collected systematically in medical records. The severity of the injuries was classified according to AIS version 2015. Both the Injury Severity Score (ISS) (Osler et al. 1997) and the New Injury Severity Score (NISS) (Baker et al. 1974) were calculated.

Length of stay in ICU/HDU was obtained from TAUH’s Trauma Register or from the medical records of individual patients.

### Statistics

Statistical analysis was performed with R version 4.0.2 (R Foundation for Statistical Computing, Vienna, Austria). Age was reported using mean (SD) and ISS was reported using median (IQR). Welch’s t-test was used to compare means. The Mann–Whitney U test was used to compare ranks between groups. A chi-square test without Yates’ correction was used to compare the proportions of trauma mechanisms between different years. Incidences between time periods were compared using incidence rate difference. Confidence interval (CI) was determined at 95%, and therefore p-values < 0.05 were considered to be statistically significant. CIs for incidence rates were calculated using Poisson’s exact method.

### Ethics, funding, and potential conflicts of interest

Under Finnish legislation, the current study is exempt from the need to obtain ethical approval because of its retrospective nature, as stated by the Regional Ethics Committee of the Expert Responsibility area of Tampere University Hospital. This study was financially supported partly by the Competitive State Research Financing of the Expert Responsibility area of Tampere University Hospital. The authors declare they have no competing interests.

## Results

During the lockdown period (March 16–May 31, 2020) 56 severely injured patients with matching inclusion criteria were treated at the 4 participating hospitals (Table 1), covering a catchment area of around 1.15 million inhabitants. Of these, 35 patients were treated at TAUH, 10 patients at KUH, 5 patients at CFH, and 6 patients at MCH. The patients were mostly men (n = 43, 77%) with a mean age of 53 years (SD 19). During the matching reference period (March 16–May 31), the number of severely injured patients treated at the participating hospitals in the years 2016 to 2018 was 173, or an average of 58 patients per year (Table 1). Of these 173 patients, 115 were treated at TAUH, 28 at KUH, 23 at CFH, and 7 at MCH. The patients were predominantly male (n = 132, 76%) with a mean age of 53 years (SD 19).

**Table 1.** Patients treated during lockdown period (March 16 to May 31, 2020) and reference period (2016–2018) at 4 hospitals. Values are frequency (%) unless otherwise specified

Factor	2020 n = 56	2016–2018 n = 173	p-value
Age, mean (SD)	47 (21)	53 (19)	0.03 **^a^**
Sex, male	43 (77)	132 (76)	0.9 **^b^**
ASA 1–2	26 (46)	79 (46)	
ASA 3–4	30 (54)	94 (54)	0.9 **^b^**
Injury severity scores, median (interquartile range)			
ISS	18 (9)	21 (10)	0.008 **^c^**
NISS	24 (10	27 (13)	0.1 **^c^**
Mechanism of injury			0.5 **^b^**
Traffic accidents	31 (55)	88 (51)	
Car	13 (23)	50 (29)	
Motorbike	7 (13)	16 (9.3)	
Bicycle	7 (13)	11 (6.4)	
Pedestrian	2 (4)	5 (2.9)	
Other	2 (4)	6 (6.8)	
Low fall (< 3 m)	13 (23)	40 (23)	
High fall (> 3 m)	8 (5)	28 (16)	
Self-inflicted (suspected)	2 (4)	0 (0)	
Assault (suspected)	2 (4)	7 (4.0)	
Blunt injury	54 (96)	166 (96)	
Penetrating	2 (4)	7 (4.0)	
Gun shot	1 (2)	1 (0.6)	
Stabbing	1 (2)	6 (3.5)	
Other	0 (0)	8 (4.6)	
Injury pattern (AIS, minimum 2)			0.9 **^b^**
Head	32 (57)	106 (61)	
Face	7 (13)	20 (2)	
Thorax	25 (45)	93 (54)	
Abdomen	17 (30)	48 (28)	
Pelvis and extremities	19 (34)	67 (39)	
External (soft tissue)	3 (5)	5 (2.9)	
Length of stay in ICU, median (interquartile range)	2 (3)	2 (4)	0.6 **^c^**

AIS = Abbreviated Injury Score.

ICU = Intensive care unit.

a Welch’s t-test.

b Chi-square test.

c Mann–Whitney U test.

Both during and before lockdown, roughly half of all injuries were traffic related (55% vs. 51%). The majority of the injuries (96%) were caused by a blunt mechanism (Table 1). The injury patterns of those patients treated before and during the lockdown period are also shown in Table 1. The rate of thoracic injuries was lower (45% vs. 54%) during the lockdown period. Otherwise, there were no notable differences in injury patterns. During the lockdown period the median ISS was 18 (IQR 9) and median NISS 24 (IQR10).

The incidence rate of severe trauma during lockdown was 6.5/10^5^ in the TAUH region, 4.1/10^5^ in the KUH region, 2.0/10^5^ in the CFH region, and 6.0/105 in the MCH region. The overall observed incidence of severe injuries throughout the whole study population (1,150,000) was 4.9/10^5^ (Table 2). The average annual reference period incidences were 7.2/10^5^ in the TAUH region, 3.7/105 in the KUH region, 2.9/10^5^ in the CFH region, and 2.3/10^5^ in the MCH region. The overall reference incidence of severe injuries throughout the whole study population was 5.1/10^5^. The change in incidence between study points was –0.2/10^5^.

**Table 2.** Number of severely injured patients (n) and incidence rate of severe injuries during lockdown (March 16 to May 31, 2020) and reference period (2016–2018) treated at 4 hospitals

	2020			2016–2018			
	n	Mean population	Incidence rate (95% CI)	n	Mean population	Incidence rate (95% CI)	Incidence rate difference (95% CI)
Total	56	1,133,274.5	4.9 (3.7–6.4)	58	1,132,671.7	5.1 (3.9–6.5)	–0.2 (–2.0 to 1.7)
Pirkanmaa Hospital District	35	536,135	6.5 (4.5–9.1])	38	531,050.5	7.2 (5.2–9.8)	–0.7 (–3.8 to 2.5)
Hospital District of Northern Savo	10	244,919	4.1 (2.0–7.5)	9	247,098.2	3.8 (1.9–6.9)	0.3 (–3.2 to 3.8)
Central Finland Hospital District	5	252,696	2.0 (0.6–4.6)	8	252,614.3	3.0 (1.4–5.7)	–1.1 (–3.8 to 1.7)
Southern Savo Hospital District	6	99,524.5	6.0 (2.2–13)	2	101,908.7	2.3 (0.6–7.0)	3.7 (–1.9 to 9.4)

CI = confidence interval

## Discussion

Despite heavy social restrictions, the incidence of severe injuries during the lockdown period (March 16–May 31, 2020) was similar when compared with earlier years (2016–2018). The incidence rate decreased 0.2/10^5^; however, our data showed a decrease of 2.0 to increase of 1.7 severely injured patients per 10^5^ inhabitants.

Several studies have been published on observed changes on traumatic injuries treated at ERs during lockdowns. However, most of these studies have concentrated on milder injuries, which are usually more common and their trends easier to follow. Interestingly, a recent article has suggested that the incidence of or the need for intensive care for traumatic head injuries in the Uusimaa Hospital District in Finland was not affected by lockdown procedures (Luostarinen et al. 2020). We are unaware of previous studies that have examined the incidence of severe injuries during the COVID-19 lockdown in a larger patient population.

Contrary to what we expected, we were not able to detect a statistically significant difference in the rate of traffic-related injuries even when there was a considerable reduction in traffic volumes on major roads. The absolute number of patients injured in traffic-related accidents was 31, which is close to the absolute annual average (29 patients per year). The high rate of traffic-related injuries can be explained by several factors. First, even in a lockdown period, people are forced to use motor vehicles for commuting. Second, as reported by the National Police Board of Finland, less traffic on less-crowded roads resulted in increased risky behavior, such as speeding and driving under the influence (DUI) (YLE News 2020). The third factor can be explained by our inclusion criteria; in order for a patient to suffer an injury serious enough to be included in the study, the accident must have had enough kinetic energy. The injury patterns were similar before and during the lockdown period. During lockdown, the number of deaths due to road traffic injuries did not increase (Statistics Finland 2020b).

It is a well-known fact that the majority of trauma patients are usually working-age males. In Finland, one part of the national lockdown policy was a countrywide recommendation directly from the President of Finland to all citizens aged 70 years and over to stay at home in quarantine-like conditions (Government Communications Department 2020a). Thus, we expected to see some reduction in the age profile of injury victims. Contrary to what we expected, the average age of severely injured patients actually increased from a mean age of 47 years to 53 years. Furthermore, 30% of patients were over 70 years of age.

It is evident that a second “wave” of COVID-19 with a rapidly increasing number of infections is currently emerging in Europe (Dong et al. 2020). Various European countries, such as France, Italy, Spain, Germany, and Belgium, have already brought in new restrictions, which bear a resemblance to those in the earlier lockdowns (BBC News 2020). Our findings suggest that the need for intensive care for severely injured patients remained unchanged throughout the lockdown period. In addition, both the length of stay in ICU and the injury severity scores were comparable before and during lockdown. The treatment of severely injured patients often requires major hospital resources that include operation rooms and ICU, which in turn may have to be re-utilized in treating patients with severe COVID-19 infection. Since the COVID-19 pandemic has increased the use of these essential resources, it could be argued that success in the acute treatment of severely injured patients can been seen as a kind of benchmark of a well-functioning healthcare system (Publications Office of the European Union 2019).

The strength of our study lies in the collaboration among four major hospitals in 4 hospital districts. Together, these 4 hospital districts provide all major trauma care for roughly one-fifth (1,150,000) of the whole population of Finland (5,518,000). Therefore, it can be assumed that these results are likely generalizable to most of the country. Also, as these hospitals are the sole providers of major trauma care in their hospital districts, it is unlikely that any major trauma was treated outside of these hospitals during the lockdown period. With the exception of the Uusimaa region, the number of COVID-19 patients treated at ICUs in Finland was fairly low (National Coordinating Office for Intensive Care 2020).

In conclusion, we did not detect a difference in the incidence of severe injuries during the lockdown period in Finland when compared with previous years. The one-third decline in traffic volumes did not reduce the number of severe traffic accidents. Even though there were small changes in patient demographics, severely injured patients do not seem to “disappear” during lockdown, even when strict restrictions are in place. Thus, stable hospital resources are needed to treat severely injured patients.

ARi conceived of the study, participated in data collection, and drafted the manuscript. VP participated in data collection, commented on the manuscript, and conducted data analysis. IK participated in data collection and commented on the manuscript. JS participated in data collection and commented on the manuscript. ARe participated in the study design and commented on the manuscript. VM participated in the design, coordination, and drafting of the manuscript.
